# The Prevalence and Serological Association of Hepatitis D Virus Genotypes in Taiwan

**DOI:** 10.3390/pathogens10101227

**Published:** 2021-09-23

**Authors:** Keva Joseph, Ciniso Sylvester Shabangu, Tyng-Yuan Jang, Chung-Feng Huang, Chia-Yen Dai, Jee-Fu Huang, Wan-Long Chuang, Ming-Lung Yu, Shu-Chi Wang

**Affiliations:** 1 M. Sc. Program in Tropical Medicine, Kaohsiung Medical University, Kaohsiung 80708, Taiwan; drkevajoseph@gmail.com; 2 Graduate Institute of Medicine, Kaohsiung Medical University, Kaohsiung 80708, Taiwan; u107567006@kmu.edu.tw; 3 Hepatobiliary Division, Department of Internal Medicine, Kaohsiung Medical University Hospital, Kaohsiung Medical University, Kaohsiung 80708, Taiwan; sls0902000@gmail.com (T.-Y.J.); fengcheerup@gmail.com (C.-F.H.); daichiayen@gmail.com (C.-Y.D.); jfliver@kmu.edu.tw (J.-F.H.); waloch@kmu.edu.tw (W.-L.C.); fish6069@gmail.com (M.-L.Y.); 4 Faculty of Internal Medicine, School of Medicine, College of Medicine, Kaohsiung Medical University, Kaohsiung 80708, Taiwan; 5 Center for Cancer Research, Kaohsiung Medical University, Kaohsiung 80708, Taiwan; 6 Center for Liquid Biopsy, Kaohsiung Medical University, Kaohsiung 80708, Taiwan; 7 Hepatitis Research Center, Kaohsiung Medical University, Kaohsiung 80708, Taiwan; 8 Department of Medical Research, Kaohsiung Medical University Hospital, Kaohsiung 80756, Taiwan; 9 Department of Medical Laboratory Science and Biotechnology, Kaohsiung Medical University, Kaohsiung 80708, Taiwan

**Keywords:** clinical significance, dual infection, HBV, HDV genotype

## Abstract

Hepatitis Delta Virus (HDV) is an RNA virus that requires the presence of hepatitis B surface antigen (HBsAg) to propagate into hepatocytes, with Genotype I being more prevalent globally. However, the prevalence of HDV genotypes in Taiwan is unknown. Accordingly, a cohort including 24 chronic HBV patients who received nucleos(t)ides (NUCs) between January 2002 and July 2018 was used to determine HDV genotypes and genotype specific serological association in chronic HBV carriers. HDV-positive genotypes in 18/24 (75%) males and 6/24 (25%) females were identified among chronic HBV patients. Viremia was lower in HDV-IV patients than in patients affected with other HDV genotypes (1.34 log_10_ copies/mL vs. 3.30 log_10_ copies/mL; *p* = 0.009). A logistics regression analysis revealed that HDV-IV was inversely proportional to HDV RNA (odds ratio [OR]/95% confidence intervals [CI]: 0.370/0.164–0.830; *p* = 0.017). The serologic association study indicated lower levels of creatinine (*p* = 0.047) and HDV-RNA (*p* = 0.009) in the HDV-IV group than the non-HDV-IV group but did not indicate any significant differences in the AST, ALT, bilirubin levels or other laboratory test factors. The three genotypes evident in Taiwan were HDV-I (4/24, 16.7%), HDV-II (6/24, 25.0%), and HDV-IV (14/24, 58.3%), and HDV-IV is the predominant HDV genotype in Taiwan. These results anticipate a clear understanding of HDV genotype serological association in chronic HBV carriers.

## 1. Introduction

The hepatitis delta virus (HDV) was discovered and detected in the nuclei of hepatocytes of patients who tested seropositive for hepatitis B virus (HBV) surface antigen (HBsAg) [1]. This virus is unique because it utilizes the hosts’ RNA polymerase and HBsAg of the HBV to propagate and then egress into liver cells and has been theorized to originate from plant viroids or/and host cell pre-mRNA through a splicing pathway [2]. The virus is approximately 35–37 nm in diameter with a small, single circular RNA genome approximating 1672–1700 nucleotides, enveloped by HBsAg—a key feature that perpetuates the ability of the virus to hijack the machinery of the hosts’ cellular functions [2,3]. It is, therefore, referred to as a defective RNA virus [4]. This notwithstanding, key modified pathways related to fibrosis, epigenetic changes, immune response, specific dysregulation of long non-coding RNA, and proteomic changes have all been suggested to promote hepatocellular carcinoma (HCC) development [5,6]. HDV has two clinical presentations: co-infection and superinfection with HBV, where co-infection is the ability of HDV and HBV to infect a host simultaneously in an acute phase, and superinfection refers to chronic HBV infection and acquiring HDV later in the clinical presentation of the disease [4].

Globally, the high prevalence of HBV has led to higher incidences of infection by HDV [7]. With vaccine intervention for HBV, it was anticipated that HDV would decline; however, due to the unpredictability and minimal knowledge on the nature of the HDV species, it has increased over the last two decades; out of 400 million HBV carriers, 5% (15–20 million) have probably been exposed to HDV [3]. Dual infection with HBV and HDV leads to aggressive progression to end-stage liver diseases. In Taiwan, chronic hepatitis B prevalence in the younger generation has decreased drastically to below 1% after the countrywide execution of the HBV vaccination program from 1986 [8,9]. HDV global prevalence ranges from 0.16–0.8% in the general population and ranges from 4.5–13% in HBV carriers [10,11], although the prevalence of HDV is decreasing and estimated to be 1.15% in the general population in Taiwan [12]. Currently, eight genotypes of HDV have been isolated based on the homology of viral nucleotide sequencing, where Genotype I is distributed globally, and others are distributed regionally. Genotypes I, II, and IV are found in Taiwan, Japan, Russia, and other Eastern Asian countries; Genotype III is more localized to the Amazon region (Peru, Colombia, Ecuador and Brazil), while Genotypes V, VI, VII and VIII are found in Africa [3].

Liver disease is complex and can manifest through a variety of pathways, from acute to severe status. Liver disease can then progress to fibrosis, liver cirrhosis, and finally total morphological changes leading to cancer, HCC. HBV/HDV coinfection increases the risk liver cirrhosis and rapid progression to HCC [13], although co-infection or superinfection increases the risk of hepatic failure in contrast to HBV infection alone [14]; regardless, HDV is not currently placed on the list of oncogenic agents along with the already enlisted HBV and hepatitis C virus (HCV). The mechanism by which HDV-related HCC occurs is not clear, and there is ongoing research on this phenomena; for example, previous research has pointed out that high levels of HDV DNA, old age, male gender, family history of HCC, serostatus of hepatitis B e-antigen (HBeAg), high serum alanine aminotransferase (AST) level, high quantitative HBsAg levels, basal core promoter (BCP) mutations, and HBV genotype C are all major risk factors in the development of liver cirrhosis [14]. Since our previous study elucidated the serial serological changes of HDV in chronic HBV patients receiving NUCs analogue therapy in Taiwan [15]. Through this study, we aim to determine the serological HDV genotypes in chronic HBV carriers and the associated factors in a cohort of the general population.

## 2. Results

### 2.1. Baseline Characteristics of HBV/HDV Superinfected Patients

The total cohort as shown in Table 1, the prevalence of HBV/HDV is higher in men than in women. The mean age of the patients was 52 years old; the mean levels of ALT, AST, and bilirubin were 211 IU/L, 226 IU/L, and 3.28 mg/dL, respectively. The mean HDV RNA level was 2.15 log_10_ copies/mL. Two patients were positive for HBeAg, and five patients were positive for anti-HCV, while mean HBsAg and HBV DNA levels were 1376.7 IU/mL and 3.58 Log_10_ IU/L, respectively. Furthermore, cirrhotic patients had significantly lower platelet (*p* = 0.003) and white blood cell (WBC) count (*p* = 0.024) than non-cirrhotic patients (Table 1). The baseline factors associated with patients with or without liver cirrhosis in HBV/HDV co-infected patients showed no significant change in HBsAg and HBV DNA levels.

### 2.2. Factors Associated with HDV Genotypes in HBV/HDV Superinfection Patients

**Figure 1:**
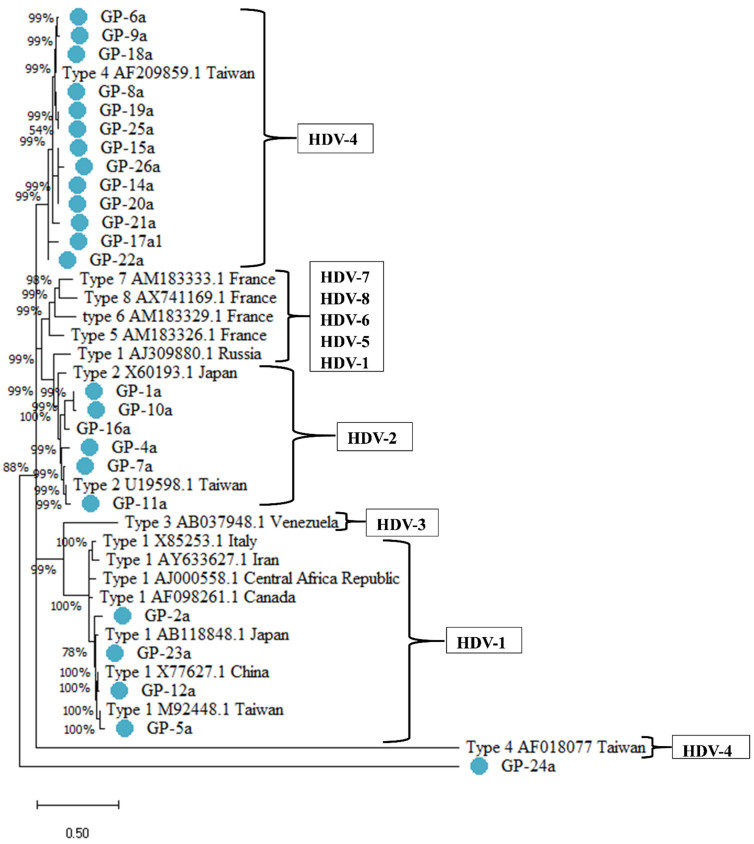
Phylogenetic analyses of HDV genomes in HBV/HDV superinfection patients in Taiwan. Phylogenetic tree was inferred using the maximum likelihood method and Kimura 2-parameter model. Taiwanese HDV sequences are referred to as “uppercase letter hyphen/number/lowercase letter”.

Specific-HDV primers were used to target highly conserved regions of the L-HDAg [16]. The first round PCR product band was 323 bp, and the second round PCR product was 234 bp by primer-specific targeting for L-HDAg (Figure S1A–D). Sample sequences retrieved from direct sequencing were aligned. The conservative sites showed unique diversity among the sample study cohort (Table S1 and Figure S2). HDV-genotyping and phylogenetic analyses revealed that the 24 patients exhibited HDV-Genotypes I, II and IV, which are associated with the general Taiwanese population (Figure 1). Of the three genotypes observed, HDV-IV (14/24, 58.3%) was predominant followed by HDV-II (6/24, 25.0%) and HDV-I (4/24, 16.7%) (Table S2). Our findings demonstrate a higher prevalence of HDV Genotype IV in the general Taiwanese cohort.

HDV viremia was significantly lower in HDV-IV genotype patients than non-HDV-IV (HDV I, II) patients (1.34 log_10_ copies/mL vs. 3.30 log_10_ copies/mL; *p* = 0.009) (Table 2). A logistics regression analysis revealed that HDV-IV was significantly inversely proportional to HDV RNA (*p* = 0.017) (Table 2); furthermore, creatinine levels were significantly lower in this group (0.96 mg/dL) than in HDV-I (2.69 mg/dL) and HDV-II (3.91 mg/dL) groups (Table 3). HDV RNA exhibited significantly lower viral loads in HDV-IV (1.34 log_10_ copies/mL) compared with those in HDV-I (3.19 log_10_ copies/mL) and HDV-II (3.34 log_10_ copies/mL) groups (Table 3). These indicate that creatinine and HDV RNA are associated with HDV genotypes in HBV/HDV superinfection patients.

### 2.3. Comparison of the Distribution of Different Biomarkers in HDV/HBV Patients with HDV-IV and Non-HDV-IV Genotype

**Figure 2:**
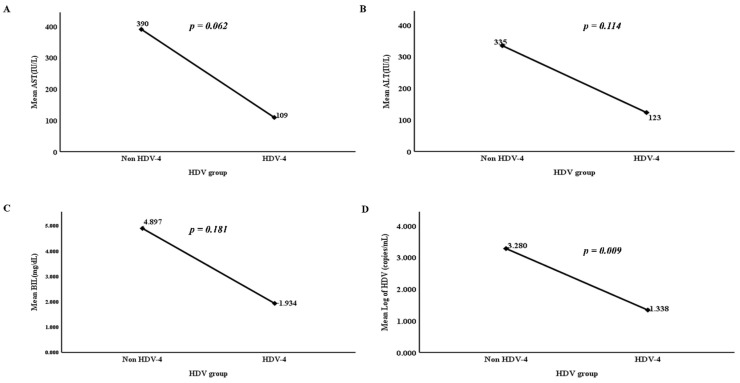
Simple linear regression graphs showing association of clinical parameters in 24 HDV/HBV superinfections. The distributions of AST (**A**), ALT (**B**), bilirubin (**C**), HDV RNA (**D**) are according to HDV genotypes. *p* values were calculated by *t*-test with *p* value < 0.05 considered to be significant.

A simple linear regression analysis between non-HDV-IV and HDV-IV genotypes was performed to observe the clinical association of biomarkers, including AST, ALT, bilirubin, and HDV RNA in the Figure 2. AST level was insignificantly associated with HDV-IV (109 IU/L) than non-HDV (390 IU/L), *p* = 0.062 (Figure 2A); ALT level was insignificantly associated with HDV-IV (123 IU/L) than non-HDV (335 IU/L), *p* = 0.114 (Figure 2B); bilirubin level was insignificantly associated with HDV-IV (1.934 mg/dL) than non-HDV (4.897 mg/dL) *p* = 0.181 (Figure 2C), while HDV RNA was significantly decreased in HDV-IV patients (1.338 log_10_ copies/mL) than non-HDV patients (3.280 log_10_ copies/mL) *p* = 0.009 (Figure 2D), demonstrating an inverse relationship between HDV-IV and HDV RNA levels. Our findings show that HDV-RNA levels may be HDV-genotype-specific in HBV/HDV superinfected patients.

### 2.4. Distribution of HDV Genotypes among Baseline Factors by Group

The HDV-IV group of patients consisted predominantly of males (55.6%), even when compared with non-HDV-IV patients (53% vs. 48%, *p* = 0.269), as shown in Table 4. From the LC group, a total of 11 patients were diagnosed, with five (38.5%) being non HDV-IV and six (54.5%, *p* = 0.729) being HDV-IV. In the ALT group with ALT levels < 400 IU/L or ≥400 IU/L, there was no significant difference (*p* = 0.139) between HDV-IV and non HDV-IV groups; however, there were thirteen (65.0%) patients with ALT < 400 IU/L and HDV-IV genotype, while three (75.0%) patients with ≥400 were dominant in non-HDV IV. In the bilirubin group with <2 mL/dL or 2 mL/dL, there was no significant difference (*p* = 0.746) between HDV-IV and non HDV-IV groups, but number of patients with bilirubin level <2 mL/dL was higher in HDV-IV. Even in the HBV DNA group with <2000 IU/mL or ≥2000 IU/mL, no significant difference (*p* = 0.746) was observed between HDV-IV and non HDV-IV groups, but there was a higher number (8, 57.1%) with HBV DNA ≥ 2000 IU/mL in the HDV-IV patient groups. Notably, seven patients were positive for HBsAg < 250 (58.3%) with HDV-IV and three patients in the range of HBsAg ≥ 250 were positive (50.0%) in both non HDV-IV and HDV-IV groups; (*p*-value = 0.737). Although there was a preponderance of the HDV-IV group in ALT ≥ 400, HBsAg < 250, and the LC group compared with non-HDV-IV, there were no significant differences.

## 3. Discussion

HDV-genotyping distribution observed in our study was consistent with that published in other notable studies in Asia [17]. Currently, eight HDV genotypes have been elucidated [18]. HDV-I, II, and IV were reported as prevalent in a few Asian countries, such as Mongolia (56.5%) [19], Pakistan (≥60%) [20], India (37%) [19], Taiwan (15%) [19], and Northern Vietnam (15.4%) [17]. A low prevalence of HDV has been observed in other Asian countries, notably Korea (0.32%) [21], Indonesia (<0.5%) [22], and the Philippines (1.6%) [18]. In our study, we observed the genotypes of patients with HDV superinfection in the general Taiwanese population. The main objective of this research was supported by the findings that the three genotypes evident in Taiwan were HDV-I (4/24, 16.7%), HDV-II (6/24, 25.0%), and HDV-IV (14/24, 58.3%). HDV-IV is predominant, followed by HDV-II in injection drug users in Taiwan even in dynamic cohorts [23].

According to a previous study, since 1986, nationwide vaccination programs for HBV have been the standard clinical practice within the Taiwanese health care system [24]. Consistency in the reports on the prevalence of HDV superinfection in patients with active hepatitis has always been estimated to be about 5% to 8%. There was a decrease in HDV superinfection from 23% in 1983 to 4.2% in 1996 among high-risk patients due to the implementation of medical and social practices, such as active preventative measures directed at promiscuity and popular use of disposable needles for drug use [24,25]. Consistent with other studies, our findings also demonstrate a decreasing prevalence of HDV in patients; however, HDV cannot be eliminated as a causative agent for liver-associated complications [12]. Sequencing and phylogenetic analysis from the region (888–1122) of L-HDVAg revealed that the Taiwanese HDV isolates belonged predominantly to HDV-IV and frequently to HDV-II, followed by HDV-I. Our findings are consistent with other studies carried out by countries within Eastern Asia [16,20,26]. The nucleotide similarity of the isolate HDV-I showed 99% identity with isolate AB118848 from Japan; HDV-II isolates showed >95% identity with U19598 from Taiwan; HDV-IV isolates showed 95% identity to AF209859 from Taiwan; while four out of fourteen isolates in HDV-IV also showed 95% identity to the AB11847 isolate of Japan, which could be due mixing of genotypes.

We were interested in the clinical significance of HDV genotypes associated with clinical factors in HBV/HDV-superinfected patients. Patient samples were categorized into non-liver cirrhosis and liver cirrhosis groups. The liver cirrhosis group had an inverse relationship with platelet counts and WBC; however, all other clinical factors revealed no significant differences in association with liver cirrhosis. HDV is associated with increased risk of liver cirrhosis and advanced liver disease [27]. HBV/HDV co-infection demonstrates lower platelet counts compared with those in HBV mono-infection, regardless of liver disease stage; however, the functional mechanism is still unknown [27,28,29], whereby when altering immune-related genes and immune response, we observed lower integrity of immune response or white blood cells in our study. In addition, patient samples were grouped into non-HDV-IV and HDV-IV groups, where lower HDV loads were observed in the HDV-IV group than the non-HDV-IV group, but few studies have focused on HDV viral loads linked to genotypes. Lastly, patients were further grouped by HDV genotypes: HDV-I, HDV-II and HDV-IV, where low levels of creatinine and HDV-RNA were observed in HDV-IV. This suggests that HDV RNA levels may be genotype-specific.

There are several limitations in the study. Firstly, when associating the HDV genotypes with the clinical (grouped) factors, there was no significance observed among the groups. This might be due to the rise in Type I errors in Chi-square tests. Secondly, the clinical parameters were not accurately retrieved from patients during follow-up, possibly owing to small sample size, absence of patient records or inconsistencies in documentation; and thirdly, it could not be determined whether patients acquired the HDV simultaneously or throughout the course of chronic HBV and HDV infections. Owing to the fact that HDV is a coinfection of virus, it is problematically complex having an HDV-only clinical infection control group; nonetheless, different HDV genotypes have been demonstrated and possible risk factors that might be genotype-specific in Taiwan were explored. Possible future studies should include in-depth research to understand how the different HDV genotypes hijack the HBV mechanism for replication, which might further unveil such genotypic specific inhibitors.

## 4. Materials and Methods

### 4.1. Study Subjects

In total, 2580 HBV Taiwanese CHB patients receiving nucleos(t)ides (NUCs) therapy between January 2002 and July 2018 at the Kaohsiung Medical University Hospital were enrolled [15]. A total of 70 patients (2.7%) were identified as being seropositive for anti-HDV. Twenty-six of the 70 patients (37.1%) in this anti-HDV seropositive population were positive for HDV RNA, making up 1.0% (26/2580 patients) of the total HBV population. Among the 26 patients seropositive for HDV RNA, two patients with missing data during follow-up were excluded. The inclusion criteria were patients positive for HBsAg and HBV DNA > 6 months, and seropositive for antibodies to HDV (anti-HDV) and HDV RNA before anti-HBV therapy, with exclusion criteria being coinfection with human immunodeficiency virus (HIV). The experiment protocol conformed to regional guidelines and was approved by the Ethics Committee of Kaohsiung Medical University Hospital (KMUH). Written consent was obtained from each patient. We defined CHB as being HBsAg-positive for at least six months or more and with chronic hepatitis delta as viral infection for a period equal to or greater than six months. A small fraction of the patients presented with LC and HCC. Liver enzymes AST and ALT, platelets (PLT), bilirubin, and other biomarkers: HBsAg, HBeAg, anti-hepatitis Be (anti-HBe) and anti-HDV were collected as clinical parameters for each patient, as shown in Table 1. Liver cirrhosis was diagnosed by histology [30], transient elastography (FibroScan; Echosens, Paris, France) > 12 kPa) [31] or through the presence of clinical, radiological, endoscopic, or laboratory evidence of cirrhosis and/or portal hypertension [32].

### 4.2. Laboratory Analyses of HBV, HCV, HDV

Hepatitis B surface antigen (HBsAg) was identified and determined through standard quantitative chemiluminescent micro-particle immunoassay (ARCHITECT HBsAg, Abbott Diagnostics) or qualitative assay (Abbott Laboratories, North Chicago, IL, USA). Hepatitis B e-antigen (HBeAg) was identified and determined using enzyme-linked immunosorbent assay kits (Abbott Laboratories). HBV DNA from the serum was determined using a standardized, automated quantitative PCR assay (COBAS TaqMan HBV test, Roche Diagnostics, Branchburg, NJ; detection limit 12 IU/mL) [33]. Anti-HDV immunoglobulin G (IgG), examined by using an anti-HDV enzyme-linked immunosorbent assay kit (General Biologicals Corporation, Taiwan) [4], was checked prior to initiating NUCs therapy, and patient serology with anti-HDV seropositivity was monitored annually from there on. HDV RNA was examined in patients seropositive for anti-HDV using a LightMix Kit HDV (Berlin, Germany) on a Roche LightCycler (detecting limit: 10 copies per mL) [18].

### 4.3. HDV Genotyping

HDV genotype was determined by direct sequencing of HDV RNA. RNA was extracted from 200 μL sera using QIAamp Kit (QIAGEN, Germany) according to the manufacturer’s protocol. Synthesized cDNA was performed in a 2-step RT-PCR protocol. The first round RT-PCR was performed using 1 μL random primer, 1 μL dNTP and 8 μL RNA, and the second round RT-PCR was performed using 2 μL DDT, 2 μL 10× buffer, 4 μL MgSO_4_, 1 μL RNase out and 1 μL SSIII RT SuperScript^®^ III (Invitrogen, CA, USA), with the synthesized cDNA being stored in aliquots at −80 °C cDNA samples from each patient sample were used as templates in the HDV-specific nested PCR to target highly conserved regions of the HDV genome. Location specificity for L-HDAg (888–1122) from reference strain NC1001653 was used to determine HDV genotypes [18]. 

The primer pairs HDV04-F (5′-GGATGCCCAGGTCGGACCG-3′) and HDV05-R (5′-AAGAAGAGRAGCCGGCCCGY’) were used in the first round PCR and primer pairs HDV06-F (5′-ATGCCATGCCGACCCGAAGA-3′) and HDV07-R (5′-GGGGAGCGCCCGGDGGCGG-3′) were used in the second round PCR (Table S1.). The PCR conditions were as follows: pre-heat temperature of 94 °C/5 min, denaturation at 94 °C/30 sec, annealing at 54 °C/45 s, extension at 72 °C/45 s, and an additional extension at 72 °C/7 min for both rounds [18]. The PCR amplification was carried out for 35 cycles, then 6–10 μL of each reaction was analyzed in 3% agarose gel electrophoresis. For the determination of patient-specific HDV isolates, 15 μL PCR products for each sample and primer were sent for direct sequencing. Here, 0.5 μL of inner sense and inner antisense primers (HDV_F/HDV_R) was used on AB 3130xl nucleic acid auto sequencer and third-generation reagent AB BigDye Terminator reagent (version 3.1) (National Cheng Kung University Center for Genomic Medicine, Kaohsiung 807, Taiwan). The generated sequences were aligned using BioEdit software 9.7 and EMBL-EBI software. A phylogenetic tree was constructed with sequenced nucleotides using Mega X software (available online: https://www.megasoftware.net, accessed on 27 August 2021). The maximum likelihood and Kimura 2-parameter model methods were employed to access maximum likelihood approach. To generate an equivalent alignment and HDV genotyping, eight prototype sequences were retrieved from NCBI GenBank (HDV-1: X77627, M92448, AB118848, NC001653, AJ000558, X85253, AY633627, AF098261; HDV-2: AJ309880, X60193, U19598, AF104624; HDV-3: AB037948, AB037949, AB037947, L22063; HDV-4: AF309420, AF018077, AB118847, AF209859; HDV-5: AM183326, AX741154, AX741159, AX741149; HDV-6: AM183332, AM183320, AJ309870, AM183329; HDV-7: AM183333, AJ584844; HDV-8: AM183330, AM183327, AX741169).

### 4.4. Statistical Analysis

Statistics are presented as mean ± standard deviations and compared using independent sample *t*-test with *p*-value < 0.05 considered to be statistically significant. A stepwise logistic regression analysis was applied to analyze factors associated with HDV genotypes. Pearson’s chi-squared test was carried out for categorical variables, while statistical analysis was performed by using IBM SPSS V26 software (available online: https://www.ibm.com/analytics/spss-statistics-software, accessed on 27 August 2021).

## 5. Conclusions

In conclusion, all 24 samples were sequenced and three HDV genotypes were found: HDV-I, HDV-II, and HDV-IV, corresponding to previous studies within the Asian region. Further studies are warranted to track the association of the clinical factors related to specific HDV genotypes. It is anticipated that through further research, a clear understanding of the HDV genotype and elevation or deceleration of liver enzymes might reveal biomarkers for the identification of HDV genotype specific liver enzymes and possibly the identification of potential genotype specific targets.

## Figures and Tables

**Table 1:** Baseline factors associated with patients with and without liver cirrhosis in HBV/HDV superinfection cohort study.

Characteristics	Total Cohort (n = 24)	Non-Cirrhosis (n = 13)	Cirrhosis (n = 11)	*p* Value
Gender (Male/Female)	18/6	1.31 (0.480)	1.09 (0.302)	0.194
Age (years, mean [SD])	52.42 (11.86)	52.92 (13.23)	51.82 (10.63)	0.826
AST (IU/L, mean [SD])	226.29 (316.23)	271.38 (356.62)	173.00 (267.64)	0.449
ALT (IU/L, mean [SD])	211.13 (321.96)	289.69 (417.81)	118.27 (106.87)	0.176
Bilirubin (mg/dL, mean [SD]) ^‡^	3.28 (4.64)	4.61 (6.21)	1.96 (1.63)	0.198
INR (mean, [SD])	1.21 (0.40)	1.10 (0.20)	1.34 (0.53)	0.183
WBC (cells/mL, mean [SD]) ^†^	6347 (2825.64)	7841 (3205.97)	5003 (1610.25)	* 0.024
PLT(x10^3^/μL, mean [SD])	157.38 (104.92)	211.77 (113.01)	93.09 (40.04)	* 0.003
Hb (g/dL, mean [SD]) ^†^	12.81 (2.06)	12.69 (1.96)	12.92 (2.58)	0.815
HBV DNA (Log_10_ IU/L, mean [SD]) ^‡^	3.81 (2.25)	3.58 (2.31)	4.08 (2.27)	0.617
HBsAg (IU/mL, mean [SD]) ^††^	1376.7 (1,616.95)	750.80 (1133.97)	2002.60 (1901.96)	0.242
HBeAg (positive/negative)	(2/22)	0.92 (0.28)	0.91 (0.30)	0.907
Anti-HCV (positive/negative)	(5/19)	0.69 (0.48)	0.91 (0.30)	0.194
HDV RNA (Log_10_ copies/mL, mean [SD])	2.15 (1.67)	2.22 (1.93)	2.06 (1.41)	0.816
Anti-HDV (mean [SD])	14.54 (7.51)	13.69 (8.56)	15.55 (6.30)	0.556

IU: international unit; INR: international normalizing ratio; HDV: hepatitis delta virus; AST: aspartate aminotransferase; ALT: alanine aminotransferase; HCV: hepatitis C virus; HBeAg: hepatitis B e antigen; data are given as mean and standard deviation [SD]; *p* value (<0.05) determined by an independent sample *t*-test and simple linear regression test; also presented for comparisons between NLC vs. LC; ^††^ n = 18; ^‡^ n = 22; ^†^ n = 19.

**Table 2:** Baseline factors associated with patients with and without HDV-IV genotype in HBV/HDV superinfected cohort study.

Variables	Non-HDV-IV (n = 10)	HDV-IV (n = 14)	*p* Value	Logistic Regression Analysis OR 95% CI *p* Value
Age (years, mean [SD])	54.10 (13.96)	51.21 (10.50)	0.568	
AST (IU/L, mean [SD])	390.30 (404.35)	109.14 (168.13)	0.062	
ALT (IU/L, mean [SD])	334.50 (403.11)	123.00 (225.44)	0.114	
Bilirubin (mg/dL, mean [SD]) ^‡^	4.90 (6.23)	1.93 (2.21)	0.181	
INR (mean, [SD])	1.31 (0.553)	1.14 (0.239)	0.32	
WBC (cells/mL, mean [SD]) ^†^	7593.75 (3,509.24)	5440.91 (1898.62)	0.102	
PLT(x10^3^ /μL, mean [SD])	170.90 (137.51)	147.71 (78.39)	0.605	
Hb (g/dL, mean [SD]) ^†^	12.90 (2.20)	12.75 (2.07)	0.877	
HBV DNA Log_10_ (IU/L, mean [SD]) ^‡^	2.35 (1.87)	1.27 (2.06)	0.298	
HBsAg (IU/mL, mean [SD]) ^††^	2467.64 (1259.46)	909.19 (1594.61)	0.175	
HDV RNA (Log_10_ copies/mL, mean [SD])	3.30 (1.82)	1.34 (0.99)	* 0.009	0.37 0.164–0.83 ** 0.017
Anti-HDV (mean [SD])	13.96 (9.24)	14.96 (6.34)	0.756	

IU: international unit; HDV: hepatitis delta virus; AST: aspartate aminotransferase; ALT: alanine aminotransferase; INR: international normalizing ratio; WBC: white blood cells; PLT: platelet count; Hb: hemoglobin; data are given as mean [SD]; * *p* value (<0.05) determined by simple linear regression test, ** *p* value determined by stepwise logistic regression; also presented for comparisons between NHDV-4 vs. HDV-4; ^††^ n = 18, ^‡^ n = 22, ^†^ n = 19.

**Table 3:** Baseline factors associated with HDV genotypes in HBV/HDV superinfection patients.

Variables	HDV-I	HDV-II	HDV-IV	*p* Value
Age (years, mean [SD]))	53.5 (9.88)	54.50 (17.09)	51.21 (10.50)	0.846
AST (IU/L, mean [SD])	443.00 (435.97)	355.17 (420.18)	119.92 (180.33)	0.086
ALT (IU/L, mean [SD])	487.75 (545.40)	232.33 (287.59)	133.83 (243.21)	0.132
Bilirubin (mg/dL, mean [SD]) ^‡^	5.74 (7.47)	4.34 (5.96)	1.93 (2.21)	0.308
Creatinine (mg/dL, mean [SD])	2.69 (3.88)	3.91 (3.65)	0.96 (0.50)	* 0.047
INR (mean [SD])	1.50 (0.84)	1.19 (0.28)	1.14 (0.24)	0.304
WBC (cells/mL, mean [SD]) ^†^	8460.00 (4919.16)	7074.00 (2924.26)	5440.91 (1898.62)	0.216
PLT(x10 ^3^ /μL, mean [SD])	196.00 (79.99)	154.17 (171.34)	147.71 (78.39)	0.734
Hb (g/dL, mean [SD]) ^†^	14.03 (1.39)	12.22 (2.44)	12.75 (2.07)	0.505
HBV DNA (Log_10_ IU/L, mean [SD]) ^‡^	3.72 (2.60)	4.50 (2.48)	3.49 (2.16)	0.686
HBsAg (IU/mL, mean [SD]) ^††^	251 (0.00)	1581 (1506)	711.73 (1340)	0.338
HDV RNA (Log_10_ copies/mL, mean [SD])	3.19 (2.22)	3.34 (1.72)	1.34 (0.99)	* 0.012
Anti-HDV (mean [SD])	12.88 (9.38)	14.69 (9.96)	14.96 (6.34)	0.895

AST: aspartate aminotransferase; ALT: alanine aminotransferase, Bil: bilirubin; INR: international normalizing ratio; WBC: white blood cell; PLT: platelet count; Hb: hemoglobin; HBV: hepatitis B virus deoxynucleic acid; HBsAg: hepatitis B surface antigen; HDV RNA: hepatitis delta virus ribonucleic acid; ^†^ n = 19; ^‡^ n = 22; ^††^ n = 18.

**Table 4:** Comparative grouped baseline factors of non HDV-IV and HDV-IV in HBV/HDV superinfected patients.

		Non-HDV-IV	HDV-IV	Total	*p* Value
Variables	Category	N (%)	N (%)	N (%)	
Gender	Male	8 (44.4)	10 (55.6)	18 (100.0)	0.269
	Female	2 (33.3)	4 (66.7)	6 (100.0)	
	Total	10 (41.7)	14 (58.3)	24 (100.0)	
LC group	Non-LC	5 (38.5)	8 (61.5)	13 (100.0)	0.729
	LC	5 (45.5)	6 (54.5)	11 (100.0)	
	Total	10 (41.7)	14 (58.3)	24 (100.0)	
ALT group	ALT < 400 IU/L	7 (35.0)	13 (65.0)	20 (100.0)	0.139
	ALT ≥ 400 IU/L	3 (75.0)	1 (25.0)	4 (100.0)	
	Total	10 (41.7)	14 (58.3)	24 (100.0)	
Bilirubin group	BIL < 2 mL/dL	6 (42.9)	8 (57.1)	14 (100.0)	0.746
	BIL ≥ 2 mL/dL	4 (50.0)	4 (50.0)	8 (100.0)	
	Total	10 (45.5)	12 (54.5)	22 (100.0)	
HBV DNA group	HBV DNA < 2000 IU/mL	4 (50.0)	4 (50.0)	8 (100.0)	0.746
	HBV DNA ≥ 2000 IU/mL	6 (42.9)	8 (57.1)	14 (100.0)	
	Total	10 (45.5)	12 (54.5)	22 (100.0)	
HBsAg group	HBsAg < 250 IU/mL	5 (41.7)	7 (58.3)	12 (100.0)	0.737
	HBsAg ≥ 250 IU/mL	3 (50.0)	3 (50.0)	6 (100.0)	
	Total	8 (44.4)	10 (55.6)	18 (100.0)	

HBV: hepatitis B virus, AST: aspartate aminotransferase; ALT: alanine aminotransferase, HBsAg: hepatitis B surface antigen, Bil: bilirubin; LC: liver cirrhosis. Results are expressed as number (%), * *p* values were calculated from Pearson’s chi-squared test and also presented for comparisons between NHDV-IV vs. HDV-IV vs. clinical groups.

## Supplementary Materials

The following are available online at https://www.mdpi.com/article/10.3390/pathogens10101227/s1, Figure S1: agarose gel electrophoresis of HDV amplicons from nested PCR using primer-specific targeting for L-HDAg. Figure S2: representative HDV sequences of Taiwan HBV/HDV superinfection patients showing patient-specific HDV isolates alignment. Table S1: primer and conditions for nested PCR carried out in this study for amplicon region of L-HDAg of HDV genome. Table S2: distribution of HDV genotypes in HBD-HDV superinfection patients.
