# Supplementary material for: The Prevalence and Serological Association of Hepatitis D Virus Genotypes in Taiwan
Source: Pathogens. 2021 Sep 23;10(10):1227. doi: 10.3390/pathogens10101227 (PMC8541235; doi:10.3390/pathogens10101227)
Supplement: Supplementary file 1 [file pathogens-10-01227-s001.zip › pathogens-1377805-supplementary.pdf]

Supplementary

Table S1. Primer and conditions for nested PCR carried out in this study for amplicon region of L-HDAg of HDV Genome.

.

HDV Genome Target Region	Primer Name	Primer Sequence (5'-3')	PCR Conditions				
			Pre-heat	Cycles	Final Extension	Product size (bp)	Reference
L-HDAg (888-1122)	HDV04-F	GGATGCCCAGGTCGGACCG	94℃/5 min	94℃/30 sec. 54℃/45 sec. 72℃/45 sec. (35 cycles)	72℃/7 min		
	HDV05-R	AAGAAGAGRAGCCGGCCCGY				323	9
	HDV06-F	ATGCCATGCCGACCCGAAGA				234	
	HDV07-R	GGGGAGCGCCCGGDGGCGG					

HDV: Hepatitis delta virus; PCR: polymerase chain reaction; bp: base pair; L-HDAg: large hepatitis delta antigen

Table S2. Distribution of HDV genotypes in HBD-HDV superinfection patients.

No.	Patient ID	Age	Gender	HDV Load(copies/ml)[1]	HDV Genotype
**1**	GP-1a	68	Male	6.0	2
**2**	GP-2a	39	Male	5.9	1
**3**	GP-4a	62	Male	4.6	2
**4**	GP-5a	56	Male	3.9	1
**5**	GP-6a	61	Male	3.5	4
**6**	GP-7a	64	Male	3.1	2
**7**	GP-8a	58	Male	2.9	4
**8**	GP-9a	45	Female	2.8	4
**9**	GP-10a	28	Male	2.7	2
**10**	GP-11a	67	Male	2.5	2
**11**	GP-12a	61	Female	2.3	1
**12**	GP-14a	39	Male	1.3	4
**13**	GP-15a	60	Male	1.2	4
**14**	GP-16a	45	Female	2.8	2
**15**	GP-17a	61	Male	1.1	4
**16**	GP-18a	53	Male	1.0	4
**17**	GP-19a	50	Male	0.8	4
**18**	GP-20a	64	Female	0.8	4
**19**	GP-21a	41	Male	0.8	4
**20**	GP-22a	31	Female	0.7	4
**21**	GP-23a	58	Male	0.6	1
**22**	GP-24a	43	Male	0.6	4
**23**	GP-25a	47	Male	0.6	4
**24**	GP-26a	64	Female	0.6	4

GP: general population patient 1a etc.; HDV: hepatitis delta virus, viral load in log_{10} copies/ml

Supplementary Figures:

Figure S1.

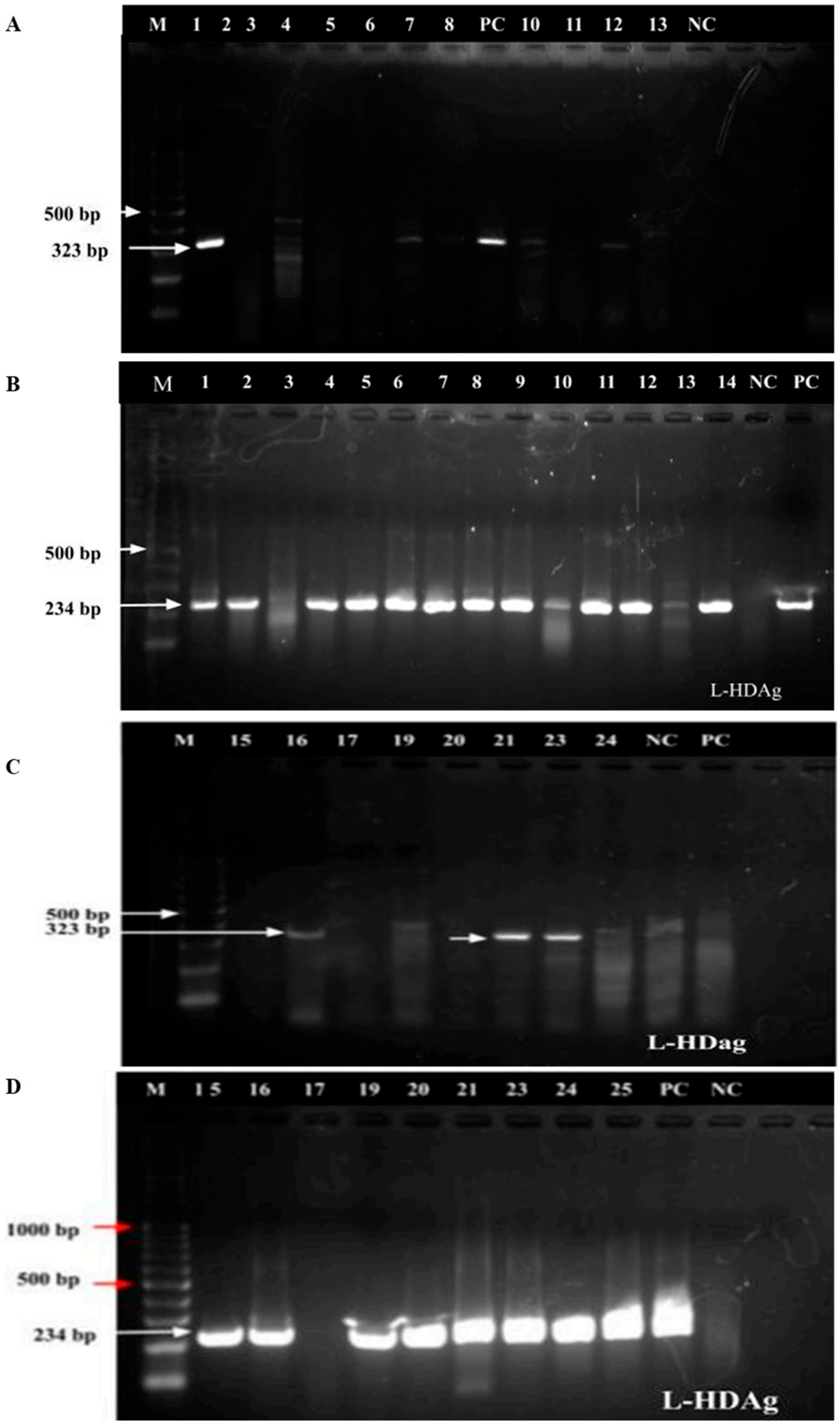

Figure S1. Agarose Gel Electrophoresis of HDV amplicons from nested PCR using primer-specific targeting for L-HDAg. 1st round PCR product is 323 bp (**A, C**), 2nd round PCR product is 234 bp (**B, D**) M: Marker; NC: negative control; PC: positive control; Samples were loaded at 6 μL (**A, B, C**); Samples were loaded at 10 μL (**D**) in 3% gel. Sample number list as below: 1: GP-1a; 2: GP-2a; 3: GP-3a; 4: GP-4a; 5: GP-5a; 6: GP-6a; 7: GP-7a; 8: GP-8a; 9: GP-9a; 10: GP-10a; 11: GP-11a; 12: GP-12a; 13: GP-13a; 14: GP-14a; 15: GP-15a; 16: GP-16a; 17: GP-17a; 19: GP-19a; 20: GP-20a; 21: GP-21a; 23: GP-23a; 24: GP-24a; 25: GP-25a.

Figure S2.

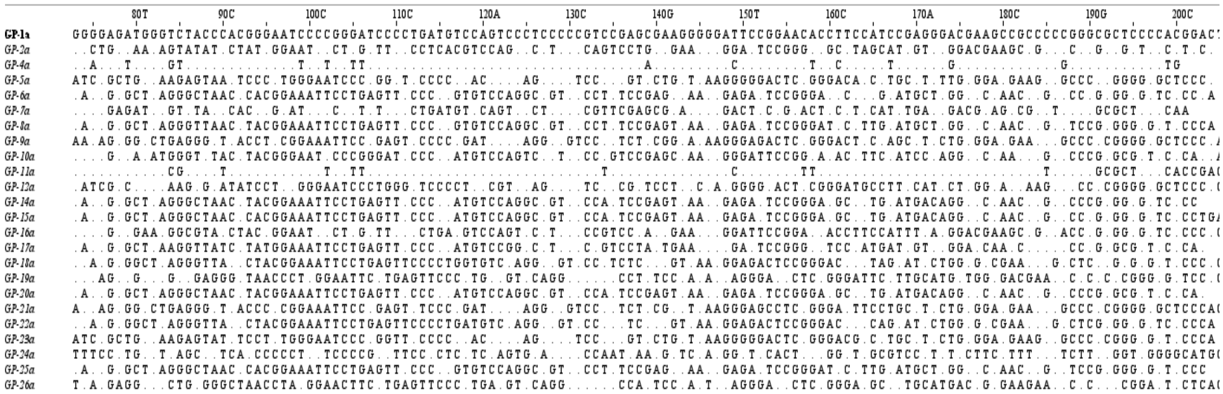

Figure S2. Representative HDV sequences of Taiwan HBV/HDV superinfection patients showing patient-specific HDV isolates alignment. Sequence table showing conserve and non-conserved regions of HDV partial genome (L-HDAg). GP-9a is used as the reference sequence for alignment. Dots depicts conservative nucleotides. Consensus graph shows the distribution pattern and color of the nucleotide in alignment. Sequence alignment was done in Jalview software v. 2.11.1.0.
